# High Fat Diet Suppresses Peroxisome Proliferator-Activated Receptors and Reduces Dopaminergic Neurons in the Substantia Nigra

**DOI:** 10.3390/ijms21010207

**Published:** 2019-12-27

**Authors:** Yu-Chia Kao, Wei-Yen Wei, Kuen-Jer Tsai, Liang-Chao Wang

**Affiliations:** 1 Institute of Clinical Medicine, College of Medicine, National Cheng Kung University, Tainan 70101, Taiwan; yukanomail2006@yahoo.com.tw (Y.-C.K.); jeffshow1010@gmail.com (W.-Y.W.); 2 Department of Pediatrics, E-DA Hospital, Kaohsiung 82445, Taiwan; 3 Research Center of Clinical Medicine, National Cheng Kung University Hospital, College of Medicine, National Cheng Kung University, Tainan 70403, Taiwan; 4 Division of Neurosurgery, Department of Surgery, National Cheng Kung University Hospital, College of Medicine, National Cheng Kung University, Tainan 70403, Taiwan

**Keywords:** high-fat diet (HFD), dopamine (DA), Parkinson disease (PD), peroxisome proliferator-activated receptor (PPAR)

## Abstract

Although several epidemiologic and animal studies have revealed correlations between obesity and neurodegenerative disorders, such as Parkinson disease (PD), the underlying pathological mechanisms of obesity-induced PD remain unclear. Our study aimed to assess the effect of diet-induced obesity on the brain dopaminergic pathway. For five months, starting from weaning, we gave C57BL/6 mice a high-fat diet (HFD) to generate an obese mouse model and investigate whether the diet reprogrammed the midbrain dopaminergic system. Tyrosine hydroxylase staining showed that the HFD resulted in fewer dopaminergic neurons in the substantia nigra (SN), but not the striatum. It also induced neuroinflammation, with increased astrogliosis in the SN and striatum. Dendritic spine density in the SN of HFD-exposed mice decreased, which suggested that prolonged HFD altered dopaminergic neuroplasticity. All three peroxisome proliferator-activated receptor (PPAR) subtype (PPAR-α, PPAR-β/δ, PPAR-γ) levels were significantly reduced in the SN and the ventral tegmental area of HFD mice when compared to those in controls. This study showed that a prolonged HFD induced neuroinflammation, suppressed PPAR levels, caused degeneration of midbrain dopaminergic neurons, and resulted in symptoms reminiscent of human PD. To our knowledge, this is the first study documenting the effects of an HFD on PPARs in dopaminergic neurons.

## 1. Introduction

Long-term changes in diet can alter the lipid composition of the brain. High fat diets (HFDs) are strongly correlated with obesity and exert detrimental effects on cognitive and behavioral functions. Epidemiologic studies have found that patients with obesity have increased risks of neuropsychiatric disorders, including anxiety and depression [1]. Meanwhile, patients with anxiety are more likely to seek palatable foods with higher carbohydrate and fat content, which predisposes them to obesity [2]. 

Epidemiologic and animal studies have also revealed correlations between obesity and neurodegenerative disorders, such as Parkinson disease (PD), Alzheimer disease (AD), and Huntington disease [3,4]. The major pathological change in PD is the degeneration of dopaminergic neurons of the substantia nigra (SN), which leads to dopamine (DA) deficiency in the striatum [5]. The main motor symptoms of PD include bradykinesia, resting tremor, rigidity, and postural instability. Many cases also present with a variety of non-motor symptoms such as anxiety, depression, hallucinations, impulse control disorders, cognitive impairment, as well as autonomic dysfunction [6]. The DA system consists of pathways that originate in the midbrain. The pathway from the pars compacta of the SN (SNpc) to the striatum is called the nigrostriatal pathway, which regulates motor control and movement, and its dysfunction causes PD [7]. The pathway from the ventral tegmental area (VTA) to the nucleus accumbens forms the mesolimbic pathway, which projects to the frontal cortex to form the mesocortical pathway. The pathway that originates from the VTA is involved in motivation, reward, emotion, and cognitive functions, and it is called the “brain reward system”; its dysfunction is related to stress, anxiety, schizophrenia, and drug addiction [8]. 

Peroxisome proliferator-activated receptors (PPARs) are transcription factors that belong to the nuclear hormone receptor superfamily. Upon activation by their ligands, PPARs heterodimerize with retinoid X receptors and then bind to PPAR response elements to regulate the expression of genes that are involved in glucose and lipid metabolism, adipogenesis, insulin sensitivity, immune responses, and cell growth and differentiation [9]. Fatty acids and eicosanoids are endogenous ligands of PPARs; therefore, dietary lipids can regulate PPAR activity [10]. There are three PPAR isoforms: PPAR-α, PPAR-β/δ, and PPAR-γ. PPAR-α plays an important role in lipid catabolism, PPAR-β/δ regulates lipid and glucose metabolism, and PPAR-γ is involved in lipid storage [11]. Apart from their pivotal role in energy hemostasis, it is documented that all three PPAR isoforms regulate inflammation [12]. 

PPARs are ubiquitously expressed in the brain, and the general order of abundance is PPAR-β/δ > PPAR-α ≥ PPAR-γ. PPAR-α is the only isotype that colocalizes with all cell types in the central nervous system (CNS), including neurons, astrocytes, and microglia, whereas PPAR-β/δ only colocalizes with neurons in the grey matter, and PPAR-γ colocalizes with neurons and astrocytes, but not microglia [13]. In the CNS, PPARs can downregulate mitochondrial and peroxisomal dysfunction, oxidative stress, and neuroinflammation. Therefore, they have been increasingly investigated for the treatment of neurodegenerative diseases [14]. We investigated whether an HFD can induce alterations in PPAR expression in the midbrain dopaminergic pathways and result in PD-like symptoms while considering the strong correlations between PPARs and lipids and the presence of all PPAR isotypes in the brain.

## 2. Results

### 2.1. HFD Induces More Body Weight Gain

**Figure 1:**
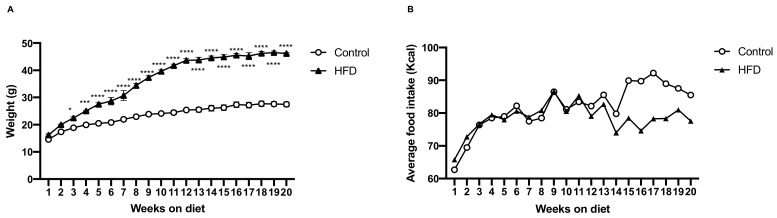
Dietary-induced changes in body weight and food intake. (**A**) After 20 weeks, high-fat diet (HFD) mice had significantly more body weight gain. (**B**) The two groups of mice with ad libitum food access had similar caloric intakes. The asterisks denote the level of statistical significance calculated using a two-way analysis of variance (* *p* < 0.05, *** *p* < 0.001, **** *p* < 0.0001); data are represented as means (*n* = 30 in each group). HFD: high fat diet.

We fed C57BL/6 mice an HFD as experimental design and standard chow as control for 20 weeks to induce diet-induced obesity (DIO). Weekly body weight and caloric measurements showed that, as compared to the control mice, the HFD mice began to significantly gain body weight from the third week (Figure 1A). Twenty weeks after the introduction of the two diets, HFD group had an 18.7% greater body weight gain than that of the control group (mean 45.8 ± 2.6 g vs. 26.3 ± 2.3 g, respectively), despite an almost similar caloric intake between HFD and control groups (Figure 1B).

### 2.2. HFD Causes Cognitive Impairment, Increased Anxiety, and Decreased Locomotor Function

We examined their motor and non-motor symptoms, specifically, cognition and anxiety, in order to verify whether the HFD could induce PD-like symptoms in mice. HFDs are known for their deleterious impact on cognition; besides, cognitive impairment is a common symptom of PD [15]. We tested the cognitive functions of HFD and control mice using the Morris water maze. 

**Figure 2:**
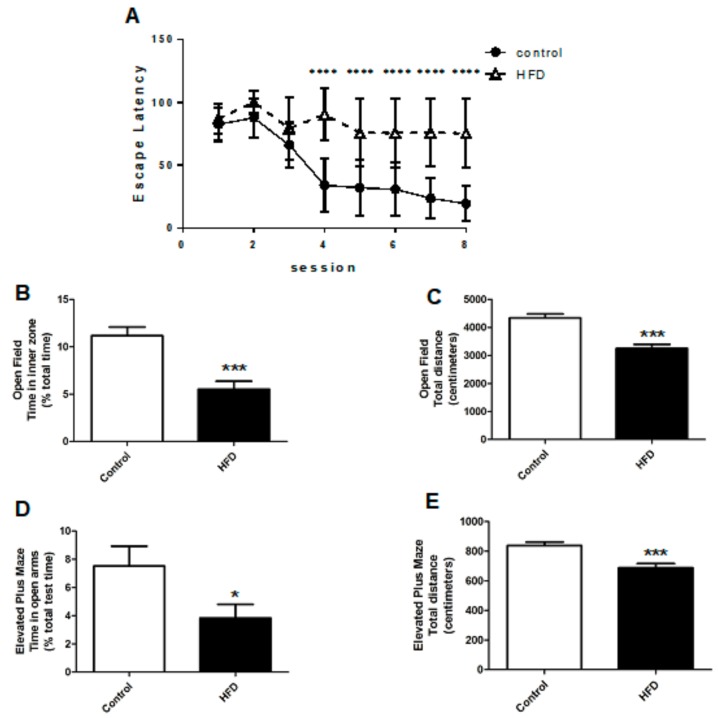
Behavioral tests for cognition, anxiety, and locomotor function in HFD and control mice. (**A**) In the Morris water maze test, HFD mice had significantly delayed escape latencies compared to the controls, indicating impaired cognition. (**B**) In the open-field test (OFT), the HFD mice spent less time in the inner zone, indicating increased anxiety. (**C**) The HFD mice traveled shorter distances compared to those by the controls in the OFT. (**D**) In the elevated plus maze (EPM), the HFD mice spent less time in the open arm, indicating increased anxiety. (**E**) The total distance traveled by HFD mice in the EPM was also less than that traveled by the controls. The asterisks represent the level of statistical significance calculated using a two-tailed Student’s *t*-test (* *p* < 0.05, *** *p* < 0.001, **** *p* < 0.0001); data are represented as mean ± SEM. (*n* = 30 in each group). OFT: open field test; EPM: elevated plus maze; SEM: standard error of mean.

In the Morris water maze, HFD mice had delayed escape latencies as compared to the control mice (82.3 ± 22.3 s vs. 47.5 ± 18.3 s, *p* < 0.0001), indicating impaired cognitive function (Figure 2A). The open-field test (OFT) and elevated plus maze (EPM) were used to examine movement and anxiety levels, as they have been validated for testing locomotor function and anxiety in animal models. In the OFT, HFD mice spent less time in the inner zone when compared to the controls, which indicated increased anxiety levels (5.5 ± 0.8% vs. 11.2 ± 0.9%, respectively, *p* < 0.001) (Figure 2B). Besides, in the OFT, the HFD mice traveled shorter distances as compared to the controls (3249 ± 142 cm vs. 4335 ± 143 cm, respectively, *p* < 0.001) (Figure 2C). In the EPM, the HFD mice spent less time in the open arm during the test as compared to the controls (3.8 ± 1.0% vs. 7.5 ± 1.4%, respectively, *p* < 0.05), revealing increased anxiety (Figure 2D). The total distance that was traveled by HFD mice in the EPM was also less than that by the controls (688 ± 28 cm vs. 839 ± 233 cm, respectively, *p* <0.001) (Figure 2E). The neurobehavioral tests strongly suggested that the HFD induced a PD-like condition in mice that presented as decreased locomotor function, increased anxiety, and impaired cognition.

### 2.3. HFD Causes Decreased Dopaminergic Neurons in the SN

**Figure 3:**
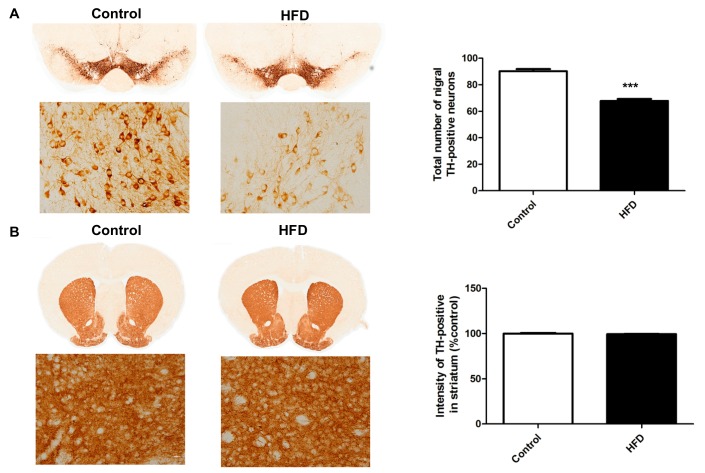
Tyrosine hydroxylase (TH) immunostaining of dopaminergic neurons in the substantia nigra (SN) and striatum. The TH-positive area was measured using ImageJ software. (**A**) HFD mice had fewer TH-positive cells in the SN compared to the controls. Upper panels, magnification 20×; lower panels, magnification 600×. (**B**) The TH-immunoreactive fiber density in the striatum was not altered between HFD and control mice. Upper panels, magnification 10×; lower panels, magnification 600×. The asterisks represent the level of statistical significance calculated using a two-tailed Student’s *t*-test (*** *p* < 0.001); data are represented as mean ± SEM (*n* = 10 in each group). TH: tyrosine hydroxylase; SN: substantia nigra.

We focused on the histological alterations in the nigrostriatal pathway, as PD mainly affects this DA pathway and is characterized by degeneration of the SNpc. Tyrosine hydroxylase (TH) is the rate-limiting enzyme in DA synthesis; hence, TH immunohistochemistry is widely used to detect DA neurons. Using TH immunostaining in the nigrostriatal pathway, we found that the percentage of TH-positive cells in the SN of HFD mice was significantly less as compared to that in the controls (Figure 3A) (67.9 ± 1.5% vs. 90.3 ± 1.6%, respectively, *p* < 0.001), indicating that the HFD could indeed reduce the number of nigral DA neurons, which is the main pathology of PD. However, the TH-immunoreactive density in the striatum was not altered between HFD and control groups (Figure 3B). 

### 2.4. HFD Causes Reduced Dendritic Spine Density in the SN and Striatum

**Figure 4:**
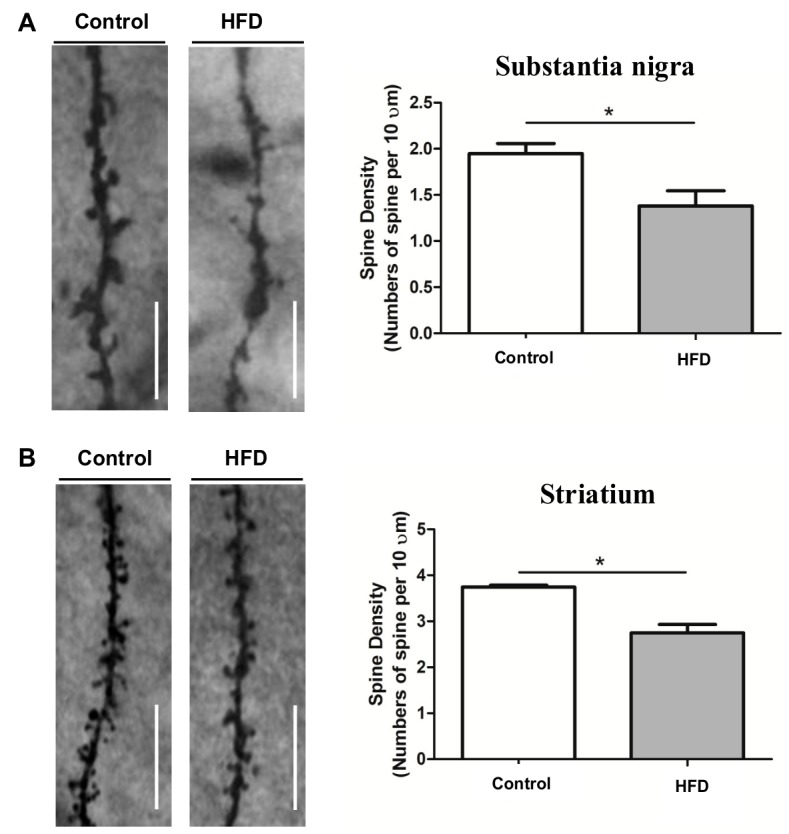
Golgi staining of dendritic spines of dopaminergic neurons in the SN and striatum. The dendritic spine density of TH-positive neurons was represented by the number of spines per 10 μm of dendritic length. HFD mice had decreased dendritic spine density in the SN (**A**) and striatum (**B**) compared to the controls. Magnification 100×, scale bar = 10 μm. The asterisks represent the level of statistical significance calculated using a two-tailed Student’s *t*-test (* *p* < 0.05); data are represented as mean ± SD (*n* = 7 in each group). SD: standard deviation.

We analyzed whether the neuronal loss was accompanied by a reduction in synaptic transmission units after documenting the reduction of DA neurons in the SN of HFD mice. We used Golgi staining to count the number of dendritic spines in the SN and striatum. Golgi staining of TH-positive neurons revealed decreased dendritic spine density in the SN (Figure 4A) (1.4 ± 0.5 vs. 1.9 ± 0.4, respectively, *p* < 0.05) and striatum (Figure 4B) (2.7 ± 0.6 vs. 3.7 ± 0.6, respectively, *p* < 0.05) of HFD mice when compared to those in the controls. Decreased dendritic spine density in these two areas is a strong evidence that the nigrostriatal DA pathway was damaged by the HFD although we did not measure the level of DA in the nigrostriatal pathway.

### 2.5. HFD Results in Increased Neuroinflammation in the Nigrostriatal Pathway

We investigated whether HFD could lead to inflammation by examining microgliosis and astrogliosis in the SN and striatum, as neuroinflammation had been proposed as an important mechanism in neurodegenerative diseases, such as PD, and obesity is associated with a chronic inflammatory state. 

**Figure 5:**
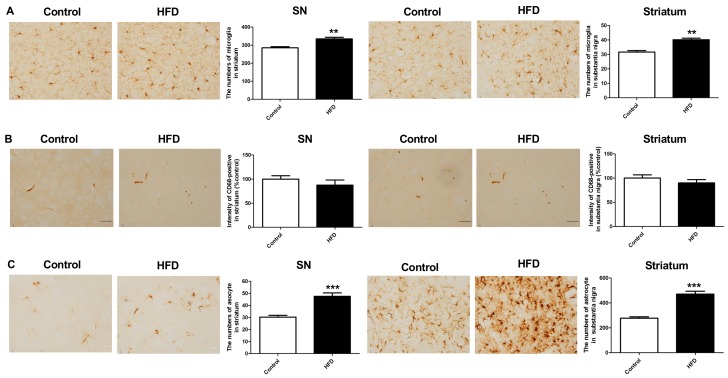
Immunostaining for astrogliosis in the SN and striatum. (**A**) We stained the microglia for Iba-1, which showed an increased number of microglia in the SN and striatum of HFD mice. (**B**) CD68 immunostaining of microglia did not show difference between HFD and control groups. (**C**) GFAP staining of astrocytes showed a significant increase in the number of GFAP-positive cells in the SN and striatum in the HFD mice compared to that in the controls. Therefore, the HFD caused increased astrogliosis in the SN and striatum. Scale bar = 100 μm. The asterisks represent the level of statistical significance calculated using a two-tailed Student’s *t*-test (** *p* < 0.01, *** *p* < 0.001); data are represented as mean ± SEM (*n* = 10 in each group). Iba-1: ionized calcium-binding adaptor molecule 1; GFAP: glial fibrillary acidic protein.

The microglia were stained for ionized calcium-binding adaptor molecule 1 (Iba-1) and CD-68, and astrocytes were stained for glial fibrillary acidic protein (GFAP). Iba-1 staining showed a significantly increase in the number of microglia in the SN and striatum of HFD mice as compared to that in controls (Figure 5A) (SN: 277.4 ± 9.6 vs. 329.4 ± 11.6, respectively, *p* < 0.01; striatum: 32.7 ± 0.7 vs. 41.8 ± 0.8, respectively, *p* < 0.01). However, CD-68 staining did not show any difference between the HFD and control groups (Figure 5B). There was a significant increase in the number of GFAP-positive cells in the SN and striatum of HFD mice as compared to that in controls (Figure 5C) (SN: 47.6 ± 2.9 vs. 30.3 ± 1.5, respectively, *p* < 0.001; striatum: 470.8 ± 22.8 vs. 277.2 ± 11.7, respectively, *p* < 0.001). Therefore, the HFD mice had more neuroinflammation in the nigrostriatal DA pathway, with an increase in the number of both microglia and astrocytes.

### 2.6. HFD Causes Reduction in All PPAR Isotype Levels in the Dopaminergic Neurons of the SN and VTA

**Figure 6:**
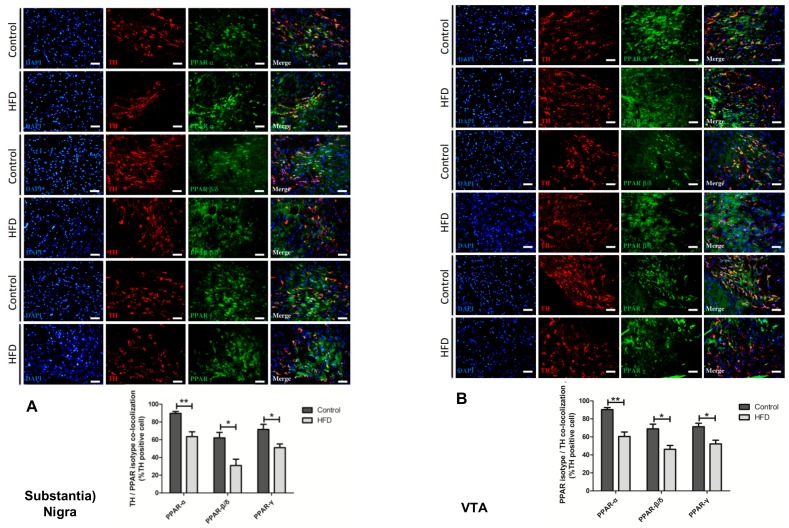
Evaluation of peroxisome proliferator-activated receptor (PPAR) isotype expression in the dopaminergic neurons of the SN and ventral tegmental area (VTA) using anti-PPAR antibodies. Immunofluorescence showed that TH neurons (second panels, coded in red) and PPAR (third panels, coded in green) were colocalized (right panels, merge images coded in yellow-orange). ′,6-diamidino-2-phenylindole was used as a counterstain (left panel, indicated by blue nuclei). Using antibodies against each PPAR isotype, levels of all the PPAR isotypes were found to be significantly decreased in the SN (**A**) and VTA (**B**) of HFD mice, compared to those in the controls. Confocal microscopy (30×), scale bar = 100 μm. The asterisks represent the level of statistical significance calculated using a two-tailed Student’s *t*-test (* *p* < 0.05, ** *p* < 0.01); data are represented as mean ± SD (*n* = 5 in each group). PPAR: peroxisome proliferator-activated receptor; VTA: ventral tegmental area.

We focused on the PPAR levels in the DA pathway, as PPARs play a pivotal role in both the development of PD and regulation of lipid metabolism, having proved that the HFD caused PD symptoms and damage in the nigrostriatal DA pathway. We used antibodies against each PPAR isotype (PAR-α, PPAR-β/δ, and PPAR-γ) to label the TH-positive neurons of the SN and VTA. Confocal microscopy showed that in these two regions, the DA neuronal population colocalizes with all PPAR isotypes. We found that, when compared to those in the controls, all of the PPAR isotype levels in the DA neurons of the SN (Figure 6A) and VTA (Figure 6B) were markedly reduced in the HFD group. The reduction of PPAR-α was most prominent (SN: 63.5 ± 10.9% in HFD mice vs. 90 ± 4.3% in controls, VTA: 60.5 ± 10% in HFD mice vs. 90.3 ± 4.6% in controls, *p* < 0.01), whereas PPAR-β/δ and PPAR-γ were also significantly decreased in the HFD group, although to a lesser degree (PPAR-β/δ of SN: 30.9 ± 14.3% in HFD mice vs. 62.1 ± 12.3% in controls, PPAR-β/δ of VTA: 46.2 ± 8.7% in HFD mice vs. 69±10.5% in controls, *p* < 0.05; PPAR-γ of SN: 51.1±8.2% in HFD mice vs. 71.5 ± 11.5% in controls, PPAR-γ of VTA: 52.2 ± 8.4% in HFD mice vs. 71.3 ± 7.8% in controls, *p* < 0.05).

## 3. Discussion

Our experiments showed that a long-term HFD downregulated PPARs, especially PPAR-α, in the SN and VTA, increased inflammation and gliosis, and decreased the dopaminergic neuronal numbers and dendritic spines in the SN. Furthermore, HFD mice presented neurological deficits of PD, such as impaired cognition, heightened anxiety, and decreased movement. The results of our experiments provide strong evidence regarding the links between HFD, downregulation of PPARs, enhanced neuroinflammation, destruction of the brain DA pathway, and the development of PD.

Krishna et al. used three HFD feeding intervals (short: 5–6 weeks, long: 20–22 weeks, and prolonged: 33–36 weeks) and found that cognitive function was unaffected by chronic HFD feeding, which is contradictory to our findings [16]. Similar to our results, prolonged HFD consumption caused mice to be hypoactive, but more anxious in another study [17]. In mice, HFD intake increases the risk of neurological disorders that are characterized by cognitive impairments, most notably AD [18]. The peripubertal period is a critical maturational window that is highly sensitive to external influences, such as that of diet on brain function [19]. The mice in our study started an HFD from weaning and continued the diet for a long period; therefore, they were greatly afflicted by the negative impacts of the HFD on cognitive function and behaviors. The correlation between HFDs and anxiety has been reported in several studies. While most studies found increased anxiety with HFD [20], some reported anxiolytic effects [21,22]. A palatable HFD was found to ameliorate anxiety and depression-like symptoms, particularly in those that were subjected to early environmental adverse events, such as maternal separation [23]. Regarding the motor aspects, several studies revealed that HFDs decreased motor function and movement in the OFT. Mice fed an HFD for 20 weeks exhibited decreased movement in the OFT and an increase in the number of missteps in a vertical grid test when compared to mice fed a normal diet [24]. Another study showed that, as compared to lean mice, DIO mice with their activity levels measured using the OFT every two weeks had fewer and slower movements beginning at week 4 and persisting through week 18 [25]. Altered mitochondrial proteins, loss of parkin, and reduction of peroxisome proliferator-activated receptor gamma coactivator 1-alpha, all of which contribute to the pathogenesis of PD, were found in the SN of HFD mice [26]. Our findings present strong evidence that the HFD caused PD-like neurobehavioral deficits as a consequence of degenerative changes in the SN, supporting the link between HFD and PD. 

The impacts of HFDs on dopaminergic pathways have also been reported in the literature. In humans, decreased DA concentration is observed with obesity [27]. Body mass index was found to be negatively correlated with 6-[^18^F]-fluoro-L-*m*-tyrosine (FMT) uptake in the dorsal caudate while using FMT positron emission tomography to detect presynaptic DA [28]. Rodent studies found a progressive decline of striatal DA function with increasing obesity [29]. DIO causes epigenetic dysregulation of the DA system. Mice continuously exposed to an HFD from weaning displayed altered DNA methylation patterns in the promoter regions of TH and of the DA transporter [30]. Several studies in human and animal models have reported that DIO decreases the basal expression of striatal DA D2-type receptors [29,31], which contributes to physical inactivity in obesity [25]. Hormones that are associated with food intake, such as ghrelin, leptin, and insulin, all have their receptors in the VTA and are associated with the DA reward system [32]; HFD strongly affects these endocrine factors. Buaud et al. reported that synaptic plasticity markers GAP-43/neuromodulin and RC3/neurogranin were both decreased in the striatum and hippocampus in HFD group [33]. As DA neurons possess a large amount of GAP-43, and decreased GAP-43 in nigrostriatal DA neurons was observed in an animal model of the presymptomatic period of PD [34], this supports our finding of HFD-induced reduction of dendritic spine density in the SN and striatum. Other proposed mechanisms linking HFD to alterations in the DA system include oxidative stress [35], mitochondrial dysfunction [36], and decreased phosphorylation of c-Jun *N*-terminal kinase [24] and Akt [37]. SN was found to be more vulnerable to changes in energy state such as glucose signaling as compared to the striatum and cerebral cortex while using db/db and HFD models [26]. Besides, the striatal TH-positive neurons are present in modest numbers in the intact striatum in monkeys and humans, but not in rodents [8,38]. This might explain why there was no difference in TH staining in the striatum between HFD and control groups in our study.

Our experiments showed increased microgliosis and astrogliosis in the SN and VTA, suggesting a pro-inflammatory effect of the HFD. It has been shown that a chronic low-grade inflammatory state characterizes obesity per se. There are several studies documenting the association between HFD and neuroinflammation. Mice provided an HFD for six months had increased numbers of white blood cells, neutrophils, and macrophage/microglia in the brain [39], which altered glial-mediated neuronal survival [40]. The activation of glial cells leads to the production of reactive oxygen and nitrogen species, initiation of phagocytosis, and upregulation of anti-proliferative and pro-inflammatory mediators. The levels of pro-inflammatory cytokines are reported to increase after starting HFD; among these, interleukin-1, interleukin-6, and tumor necrosis factor- α [41,42,43] are the most frequently reported. Increased chemokine expression (chemokine (C-X-C motif) ligand 1 and chemokine (C-C motif) ligand 3 (CCL3)) [39] and toll-like receptor-4 signaling pathway (inhibitor of nuclear factor κB -α (IκB-α) and nuclear factor κB) [44] have also been mentioned. All of these contribute to neuronal damage and death. It seems that neuroinflammation that is induced by HFD is long-lasting, as one study that introduced only 14 days of overfeeding to neonatal rats found microgliosis in the hippocampus, which persisted into adulthood [45]. Our study showed that the HFD increased Iba-1 immunoreactive cells in the SN and striatum, indicating microgliosis in these areas. However, CD68 immunoreactivity, indicating phagocytic activity, was not increased. Microglial activation has been shown to play an import role in the pathology of PD. Chemoattractants that were released by the dying DA-activated microglia even prior to the death of nigral DA neurons and microgliosis were in parallel with neuronal dysfunction and loss of DA terminals. However, an increase of CD68 immunoreactivity is related to the duration of PD, with levels that are significantly higher in cases of shorter disease duration [46]. This suggests that microglial phagocytosis with CD68 expression might not persist when it is no longer functionally relevant. In our present study, the long-term HFD may have caused chronic insidious loss of DA neurons and terminals, such that CD68 expression was not significantly increased. 

Our data showed decreased PPAR levels in the SN and VTA of HFD mice. Dysregulation of lipid metabolism affects the expression of PPARs. PPAR-α and its target gene carnitine palmitoyltransferase 1 (CPT-1) have a pivotal role in fatty acid oxidation in mitochondria, and the PPAR-α/CPT-1 pathway is important in the inhibition of DIO. HFD can recruit PPAR-α and stimulate hepatic CPT-1A [47]. However, our study found a reduction in PPAR-α levels in the SN and VTA in HFD mice. Brain PPAR-γ is associated with obesity. Blocking CNS PPAR-γ using its antagonists or short hairpin RNA led to a negative energy balance and restored leptin sensitivity in HFD-fed rats [48]. In the brain, HFD feeding for 12 weeks in mice resulted in an elevation of peroxisomes and PPAR-γ within the hypothalamus [36]. However, another study showed that, when compared to that in the diet-restriction group, the hypothalamic PPAR-γ level was decreased after five weeks of HFD, but there was no change in the PPAR-γ level after a prolonged HFD of up to 21 weeks [49]. The contradictory results regarding the association between HFDs and hypothalamic PPAR-γ levels remain to be explored. HFD induced alterations of retinoid (retinoid acid receptor and retinoid X receptor) and PPAR signaling pathways in the striatum, with decreased PPAR-δ levels and increased retinoid receptor RXR-βγ mRNA levels, but decreased RARβ mRNA levels when compared to those in the control group. The above studies provide evidence that HFDs may modulate the expression of PPARs. The modulation of PPAR function might be associated with HFD-induced neuronal effects. In the hypothalamus and forebrain, PPAR-δ protects against the neuronal effects of HFDs [50]. Neuron-specific deletion of PPAR-δ led to leptin insensitivity and increased susceptibility to DIO in mice. However, these mice were resistant to diet-induced elevation in CNS inflammation and lipid accumulation, which disproved the concept that PPAR-δ activation inhibits lipid accumulation [51]. On the other hand, the deletion of PPAR-γ in the brain, specifically in the pro-opiomelanocortin neurons, resulted in resistance to DIO. The loss of PPAR-*γ* in neurons in male mice reduces their food intake and body weight gain when fed an HFD and abolishes leptin resistance [52]. Surprisingly, in brain PPAR-δ and PPAR-γ depleted mice, the levels of peripheral inflammatory mediators, such as circulating cytokines, and tissue inflammatory gene expression were not significantly different from that in wild type mice after being given an HFD [51,53]. 

Dysregulation of PPARs is associated with the development of PD. PPAR-α is expressed by DA neurons of the nigrostriatal circuit [54]. PPAR-α null mice, despite normal locomotion in the OFT, had reduced numbers of DA neurons in the SN, which suggested that PPAR-α is necessary for the normal development of the SN [55]. PPAR-α agonist fenofibrate and palmitoylethanolamide could prevent DA cell death in the SNpc, attenuate the loss of TH immunoreactivity in the striatum, and reverse motor deficits by 1-methyl-4-phenyl-1,2,3,6-tetrahydropyridine (MPTP) [56,57] or 6-hydroxydopamine (6-OHDA) [58]. PPAR-α agonist fenofibrate, PPAR-γ agonist pioglitazone, and PPAR-α/γ dual agonist 2-[4-(5-chlorobenzo [d] thiazol-2-yl) phenoxy]-2-methylpropanoic acid (MHY908) could also protect against DA neuronal loss, motor deficit, depression-like behavior, and the impairment of learning and memory caused by MPTP [59,60,61]. PPAR-β/δ is expressed in the nuclei of DA neurons and in astrocytes. Mice null for both PPAR-δ and PPAR-γ receptors showed the lowest levels of TH-positive cells following MPTP administration, whereas the presence of one or both of these receptors shows a trend toward protection against this degeneration [62], which suggests the importance of PPAR-β/δ in the development of PD. PPAR-β/δ agonist GW0742 could reduce the loss of DA neurons and increase the locomotor activity in MPTP or rotenone model of PD by suppressing ER stress [63,64]. 

In nonhuman primates, PPAR-γ expression is prominent in the subthalamic nucleus, oculomotor nucleus, VTA, and, to a lesser extent, in the putamen [65]. PPAR-γ agonists pioglitazone and rosiglitazone have anti-inflammatory and anti-oxidative properties [66]. PPAR-γ agonists pioglitazone or telmisartan and partial agonist GW855266X could protect against MPTP or 6-OHDA-induced loss of DA neurons in the SN and the depletion of striatal DA [67,68,69]. Oral pioglitazone administered to rhesus monkeys could cross the blood brain barrier to ameliorate the parkinsonian syndrome, attenuate both CD68-positive microglia/macrophage, and loss of both striatal terminals and nigral neurons after the injection of MPTP [70]. Therefore, PPAR-γ agonists could enhance mitochondrial function, attenuate oxidative stress, restore striatal DA, and reduce nigral microglial activation [71]. The aforementioned studies proved that all PPAR isotypes play important roles in the regulation of the dopaminergic pathway. Since PPARs act as lipid messengers to regulate metabolic homeostasis and modulate inflammation, it is reasonable that all PPARs in the midbrain DA neurons are affected by HFD, and PPAR-α, which is the key PPAR in lipid metabolism, is affected the most. DIO is known for its linkage to neurodegeneration, but little is known about the effects of HFDs on PD. Our study provides strong evidence in this regard and for the association between HFD and PD. 

Nevertheless, there were several limitations in our present study. In our study, by only using the immunostaining, there is no further mechanistic link between HFD and PD-related factors observed in the brains. Nor was cellular secondary signaling factors studied in the PPRA pathway. We only examined the DA neurons using TH staining, but did not measure the amount of DA or the change in DA transporters or receptors, which are of paramount importance for DA function. We did not use agonists or antagonists targeting each PPAR isotype to verify whether these PPAR ligands can reverse or cause the neuronal loss and behavioral effects induced by the HFD, although the study proved that all PPAR isotypes are involved in HFD-induced dopaminergic neuronal loss. Besides, whether the reduction of all PPAR levels in dopaminergic neurons after a long-term HFD is related to neuroinflammation with secondary degeneration of DA neurons in the midbrain or directly related to the lipotoxic effects of the HFD is still not known and it requires further investigation.

## 4. Materials and Methods 

### 4.1. Animals and Diets

Male C57BL/6J mice were housed in the animal center of the National Cheng Kung University (NCKU), Tainan, Taiwan. The experimental procedures for handling the mice were in accordance with the guidelines of the Institutional Animal Care and Use Committee (IACUC) of the NCKU and the National Cheng Kung University IACUC approved (2017/106011) the study. The mice were individually maintained under standard conditions (22 °C, 12 h light/dark cycles). The mice were randomly divided into two groups and fed for 20 weeks, as follows: The control group was fed a standard rodent chow (3 kcal/g) and the experimental group was fed an HFD consisting of standard rodent chow supplemented with 60% fat content (60% kcal fat, Research Diets, D12492) (*n* = 30 for each group). All mice had ad libitum access to their feed and drinking water (0 kcal/g). The feeds contained standard vitamins and minerals mixed with all essential nutrients and they were provided in powder form. Body weight and food and water consumption were measured weekly.

### 4.2. Neurobehavioral Tests

#### 4.2.1. Morris Water Maze

The effect of diet on cognitive function was assessed using the Morris water maze when the mice were around six months old. A swimming pool (180 cm diameter, 50 cm depth) contained an escape platform (10 cm diameter) submerged 4 cm beneath the water. Visual cues, such as colored shapes, were placed around the pool in plain sight of the animal. The mice were trained in the water maze with four consecutive trials per session. A total of 24 sessions were conducted over six days. When released, the mouse swam around the pool in search of an exit, and in subsequent trials the mouse was able to locate the platform more rapidly. Each trial lasted until the mouse found the platform or for a maximum of 2 min. Escape latency was defined as the average time that each mouse spent to reach the platform and was recorded while using a stopwatch.

#### 4.2.2. Open-Field Test (OFT)

The mouse was placed in a white square open-field arena (100 × 100 cm) that was enclosed by 40-cm-high walls and exposed to strong illumination (200 lux). The arena was divided into 25 squares (20 × 20 cm) consisting of nine central (the so-called inner zone) and 16 peripheral squares. Each mouse was placed in the open-field arena for 5 min. for habituation, followed by a 30 min. recording for analysis. The total distances moved and the duration of movement were monitored while using a Etho Vision XT 5.1 software (Noldus information technology, Wageningen, The Netherlands) 

#### 4.2.3. Elevated Plus Maze (EPM)

The EPM consisted of two open arms and two closed arms (50 × 10 × 36 cm) that were connected by a central platform (10 × 10 cm). The maze was elevated 60 cm above the floor and illuminated by a 100-watt light bulb fixed 2 m above the maze floor. During the test, each mouse was placed on the central platform of the maze with its head facing an open arm. The data on the total time spent in the open arms and the total distance moved were collected over a period of 7 min.

### 4.3. Brain Tissue Preparation

After the neurobehavioral tests were complete, the mice were sacrificed when they were around 26 weeks old. They were anesthetized using pentobarbital (60 mg/kg, intravenous) and transcardially perfused with phosphate-buffered saline (PBS). After decapitation, the brains were removed, fixed in 4% paraformaldehyde, and then cryoprotected by immersion in a graded (10–40%) sucrose/0.1 M PBS, pH 7.2 solution. 10 μm thick serial coronal brain sections were prepared while using a freezing microtome.

### 4.4. TH Staining

TH immunostaining was used to demonstrate the distribution of dopaminergic neurons. Sections from the SN and striatum were selected. Free-floating brain sections were incubated overnight at 4 °C in rabbit anti-TH antibody (1:200, AB152, EMD Millipore, Burlington, MA, USA), followed by 1 h in biotinylated goat anti-rabbit secondary antibody (1:500, NEF812001EA, PerkinElmer, Waltham MA, USA). Immunoreactivity was visualized by incubating the sections in a DAB Substrate-Chromogen solution (DAB; K3468, Dako Cytomation) for 3 min. The sections were washed three times with PBS and then mounted onto gelatin-coated slides. The number of TH-positive cells in the SNpc was counted while using a light microscope (LSM510; Carl Zeiss, Jena, Germany). TH-immunoreactive fiber density in the striatum was measured using TissueFAXS and analyzed while using TissueQuest (Tissue Gnostics, Vienna, Austria).

### 4.5. Immunohistochemistry for Neuroinflammation

Microglial cells and astrogliosis in the SN and striatum were assessed using Iba-1, CD68, and GFAP immunostaining. Endogenous peroxidase activity was neutralized by a 20 min. incubation in 0.3% H_2_O_2_. After washes in PBS containing 0.05% Triton-X (dilution media), background staining was blocked by a 1 h incubation in a Tris buffered saline solution containing 3% normal horse serum, 2% bovine serum albumin, and 0.05% Triton X-100. The sections were then incubated with the required primary antibody: anti-GFAP (Millipore, 1: 1000), anti-Iba1 (Chemicon, 1:500), or anti-CD68 (Chemicon, 1:300). After washes in dilution media, the sections were placed in the avidin biotin (Elite ABC kit, Vector Laboratories, Burlingame, CA) substrate (1:1000) for 75 min. The sections were then washed in 0.1 M imidazole/1.0 M acetate buffer, pH 7.4, and allowed to react in a DAB Substrate-Chromogen solution (K3468, DAKO, Carpinteria, USA). Nickel sulfate was added to the DAB chromogen reaction. Immunoreactivity was measured while using TissueFAXS and analyzed using TissueQuest (Tissue Gnostics, Vienna, Austria).

### 4.6. Golgi Staining for Dendritic Spines

For Golgi staining, the brain sections were immersed in Golgi solution containing a mixture of potassium dichromate, mercuric chloride, and potassium chromate dissolved in 5% bidistilled H_2_O in the dark for 10 days. The brains sections were washed with bidistilled-H_2_O and then transferred to a bottle containing 30% sucrose solution at 4 °C in the dark. Brains were cut into 200 μm thickness coronal sections using a vibratome (Dosaka; Japan). After drying, the slides were placed in the staining racks and washed sequentially with distilled water twice for 5 min., 50% ethanol for 5 min., 3: 1 ammonia solution for 8 min., distilled water twice for 5 min., 5% sodium thiosulfate for 10 min., distilled water for 1 min., 70%, 95%, and 100% ethanol for 6 min. each, and xylene for 6 min. For mounting, five drops of Eukitt (quick-hardening mounting medium; 03989, Fluka Analytical, Munich, Germany) were added, and the slide was then covered with cover glass. The dendritic spines were focused on under high magnification (40× and 100×) while using an Axio Imager D2 (Zeiss Instruments, Inc., Oberkochen, Germany) in order to count spine numbers and specifically, for measuring dendritic spine density, expressed as the average number of spines per μm of dendritic length. The total dendritic length and branch numbers were measured while using the ImageJ software.

### 4.7. Immunofluorescence Staining for PPARs

Immunofluorescence to characterize PPAR expression in brain sections was performed using sequential day staining. After 3 × 10 min. washes in PBS, nonspecific binding was blocked by incubating for 1 h at room temperature in a blocking buffer containing dilution media, 5% normal serum, and 0.5% Tx-100. To assess colocalization of PPARs and TH, after washing and blocking, the sections were first incubated overnight at 4 °C with the primary antibodies—anti-PPAR-α (1:100;11540-1-AP, Proteintech), anti-PPAR-β/δ (1:100;1056-2-AP, Proteintech), or anti-PPAR-γ (1:100;16643-1-AP, Proteintech), and anti-TH (AB152, EMD Millipore). For the secondary antibody-antigen reaction, the tissues were rinsed in PBS and then incubated in Alexa Fluor 488-conjugated donkey anti-rabbit antibody (1:300; Invitrogen) and Alexa Fluor 594-conjugated goat anti-mouse antibody (1:300; Invitrogen) for 1 h at room temperature. The quantification of PPAR positive cells was performed while using the Olympus BX-51 microscope. PPAR isotype and TH dual-labeled cells were quantified in the SN and VTA.

### 4.8. Statistical Analysis

All of the statistical analyses were performed using the SPSS software (SPSS, Chicago, IL, USA). The Morris water maze data were analyzed using a two-way analysis of variance. The mean values were analyzed using a two-tailed Student’s *t*-test. Results are represented as mean ± standard deviation or mean ± standard error of the mean and were first examined while using an *f*-test to determine the homogeneity of variance. Statistical significance was set at *p* < 0.05 and it is presented as * *p* < 0.05, ** *p* < 0.01, *** *p* < 0.001, **** *p* < 0.0001.

## 5. Conclusions

This study demonstrated that the mice had cognitive impairment and increased anxiety levels and exhibited motor impairment after chronic HFD feeding for five months. The motor and behavioral manifestations were similar to those seen in patients with PD. Regarding the nigrostriatal dopaminergic pathway, the HFD resulted in decreased DA neurons and dendritic spines in the SN, but no significant reduction of DA neurons in the striatum. The HFD also resulted in neuroinflammation with increased astrogliosis. Finally, we proved that in the SN and VTA of prolonged HFD-fed mice, all of the PPAR isotypes were significantly decreased. Our present study highlights the linkages between HFD, dysregulation of PPARs, neuroinflammation, and PD, although the detailed mechanisms remain to be explored.

## Abbreviations

MDPI	Multidisciplinary Digital Publishing Institute
DOAJ	Directory of open access journals
TLA	Three letter acronym
AD	Alzheimer disease
HFD	High fat diet
DA	Dopamine
DIO	Diet-induced obesity
EPM	Elevated plus maze
OFT	Open field test
PD	Parkinson disease
PPAR	Peroxisome proliferator-activated receptor
SN	Substantia nigra
TH	Tyrosine hydroxylase
VTA	Ventral tegmental area
